# Bovine milk and milk protein– promotor or inhibitor of bacterial biofilm formation at the tooth surface?

**DOI:** 10.1186/s12903-025-06432-1

**Published:** 2025-07-02

**Authors:** A. Kensche, C. Pohl, S. Basche, A. Dürasch, T. Henle, M. Hannig, C. Hannig

**Affiliations:** 1 Clinic of Operative and Pediatric Dentistry, Medical Faculty Carl Gustav Carus, TU Dresden, Fetscherstr. 74, D-01307 Dresden, Germany; 2 Chair of Food Chemistry, TU Dresden, Dresden, Germany; 3 Clinic of Operative Dentistry, Periodontology and Preventive Dentistry, University Hospital, Saarland University, Building 73, D- 66421 Homburg/Saar, Germany

**Keywords:** Pellicle, Bovine milk, Casein, Bacteria, Fluorescence microscopy

## Abstract

**Background:**

The present study aimed to investigate if mouthrinses with different types of bovine milk or milk protein isolates influence the initial bacterial colonization of the tooth surface.

**Methods:**

From 8 subjects, different biofilm samples were collected in situ on bovine enamel slabs: after 3 min of pellicle formation, mouthrinses with homogenized UHT-milk (0.3% and 3.5% fat), homogenized fresh milk (3.5% fat), non-homogenized milk 3.8%, 30% UHT-cream or a 3% micellar casein isolates containing preparation were performed, followed by a continued intraoral slab exposure for 8 h overnight. As control, no rinse was adopted. Afterwards, bacterial adhesion was quantified by DAPI staining and bacterial viability was determined by BacLight LIVE/DEAD-staining. Extracellular polysaccharides were visualized by Concanavalin A/Alexa-Fluor 594-staining. Statistical analysis was performed by the Kruskal-Wallis test and the Mann-Whitney U test followed by Bonferroni-Holm correction.

**Results:**

After 8 h of intraoral biofilm formation, 1.62*10^6^±1.68*10^6^ bacteria/cm^2^ were quantified in the control samples. Viability staining showed a distribution of 35% vital to 65% avital bacteria. None of the applied mouthrinses showed a significant change (*p* > 0.01) in bacterial colonization. A tendency to reduce bacterial colonization in situ was observed for non-homogenized milk and casein micelles.

**Conclusion:**

Mouthrinsing with bovine milk and milk protein isolates had no significant impact on initial biofilm formation at the tooth surface. Clearly, it does not increase bacterial colonization.

## Background

Nowadays, more and more researchers in the field of dentistry and nutrition medicine dedicate their interest to the investigation of potential effects of specific nutrients on biofilm formation and oral health. There are increasing numbers of scientific studies that reveal the high potential of nutritional components and dietary behaviours to reduce the occurrence or the progression of the biofilm related oral pathologies caries and periodontitis [1–6]. Different in situ studies have shown that food components such as polyphenols or fatty acids have a significant impact on the density and composition of bacterial biofilms at the tooth surface [1, 3, 7–9]. It is therefore understandable that the potential modification of bioadhesion processes at the tooth surface by common food components has become a reasonable research topic. Especially since we know that approximately 3.5 billion people are still affected by oral diseases worldwide [10, 11].

In this context, the effects of bovine milk on oral health are also under critical review [4, 12–16]. Bovine milk has repeatedly been discussed as a caries-preventive food [4, 16–19]. Besides the high levels of calcium and phosphate, a positive impact of bovine milk on the prevention of caries could especially be linked to caseins [4, 13, 20]. With 80–87%, the casein group is the main protein fraction in bovine milk [16, 21]. Based on their serine phosphate residues, these are divided into the richly phosphorylated, calcium-sensitive 𝛼_S1_-, 𝛼_S2_-, and β-caseins and the calcium-insensitive 𝜅-casein [21, 22]. Due to high contents of structure-breaking proline, caseins lack a stable secondary structure, meaning that hydrophobic amino acid residues are exposed and determine the character of the caseins [21]. Only 𝜅-casein exhibits an amphiphilic character due to its hydrophilic C-terminus. The casein micelles in bovine milk are stabilized by calcium phosphate, hydrogen bonds and hydrophobic interactions [21, 23].

According to the literature, casein molecules might have an effect on metabolic processes of caries-associated oral bacteria: in the presence of 𝜅-casein, a reduced enzyme activity of bacterial glycosyltransferase was observed in vitro and in situ [15, 19]. Furthermore, the adhesion of oral bacteria at the tooth surface could be altered by casein-containing bovine milk. A decreased bacterial adhesion by 𝜅-casein or caseinoglycomacropeptide was shown in some, but not in all in vitro studies [24–26]. Also, decreased adhesion of *Streptococcus mutans* and *Streptococcus sobrinus* has been shown in vitro [17, 27, 28]. However, the exact mechanisms of bovine milk and dairy products in the oral cavity have been studied little so far.

In situ, any (patho) physiological processes at the tooth surface involve the pellicle [29]. Therefore, alterations of pellicle formation must be investigated when studying a substance’s potential impact on caries prevention [12, 13, 20, 30]. On the one hand, the in situ pellicle provides antibacterial enzymes [31, 32]. On the other hand, it exposes the obligatory receptor sites for bacterial adhesion [29, 32–34]. Interestingly, the micellar caseins contained in bovine milk show structural similarity to protective salivary proteins involved in the pellicle formation [13, 29, 35–38]. Relevant indications for an incorporation of casein micelles into the pellicle structure were found in situ after rinsing with different types of bovine milk [13, 20]. These studies also showed, that the fat-content and the processing of bovine milk (homogenization and heating) have a relevant impact on the extend of casein-derived pellicle modifications [13, 20].

So far, no reliable in situ studies have investigated the impact of the respective pellicle modifications on bacterial biofilm formation *in situ.* One assumption could be that the adsorption of casein micelles at the tooth surface competes with the generation of bacterial receptor sites. This would hamper the adhesion of cariogenic bacteria and glycosyltransferases to the pellicle. As a result, glucan formation and thus plaque growth should be reduced [15–17, 28]. The present study aimed to investigate the influence of different commercial types of bovine milk and an experimental rinsing solution with pure casein micelles on the early bacterial colonization of the in situ pellicle. Types of bovine milk that have previously been confirmed to have an influence on the pellicle ultrastructure and the incorporation of caseins into the pellicle were selected for the investigation [13, 20]. It was expected that the application of all types of milk and milk protein isolates would alter the density and the viability of the initial bacterial biofilm adhering to the in situ pellicle. Specifically, the authors of this study hypothesized that bovine milk’s protein components would induce an impediment of bacterial colonization at the tooth surface.

## Methods

### Subjects and specimens

The present investigation was designed and performed referring to similar previous in situ studies, which likewise investigated the effects of (food) components on initial biofilm formation at the tooth surface [9, 39–43].

Bacterial biofilm formation was investigated on bovine enamel slabs that were exposed to the oral cavity for 8 h over night. Eight healthy volunteers (4 female, 4 male, aged 25–50) took part in the investigation. They were members of the laboratory staff or dentistry students. The intraoral exposure of all materials and experimental mouthrinses was approved by the ethics committee (vote: EK 147052013; Medical Faculty, Technische Universität Dresden, Germany) and the volunteers had given their informed written consent about participation in the study. A careful examination at baseline by an experienced dentist assured that neither of them had any unrestored carious lesions, periodontal diseases (PBI ≤ 12%, PSI ≤ 2) or a limited salivary flow (anamnestic or clinically visible dry mouth, dull mucosa). All volunteers confirmed to be neither sick nor in reconvalescence after disease. They were all non-smokers, took no medication and they were free of any medical history of milk allergies. The volunteers were instructed to avoid an additional use of chemical oral care products other than toothpaste during the whole study period.

Individual upper jaw splints were prepared for every participant with little cavities in the buccal regions of the teeth 15 and 16 as well as 25 and 26. During every in situ experiment, four cylindrical bovine enamel slabs (ø 5 mm) were fixed in these cavities with polyvinyl siloxane impression material (Provil novo light regular set, Heraeus Kulzer, Germany), so that only one side would be exposed to the oral fluids.

All enamel slabs were prepared from the labial surfaces of bovine incisor teeth of 2-year old cattle (BSE-negative) as already described multiple times before [9, 13, 44–46]. The bovine incisor teeth were obtained as a side product from bovine heads after their normal processing in a slaughterhouse, no animals were sacrificed for the study’s purpose (Schlachthof Emil Färber GmbH & Co. KG, Freiburg im Breisgau). The adoption of bovine enamel for in situ experiments was checked and approved by the veterinary department of the Saxon State Directorate (Veterinärwesen Landesdirektion Sachsen).

The surfaces of the enamel slabs were wet-ground and progressively polished with up to 4000 grit abrasive paper. The resulting smear layer was removed by steam jet and ultrasonication (US) with 3% NaOCl for 3 min followed by washing the specimens twice for 5 min in distilled water activated by US. Afterwards the bovine enamel slabs were disinfected in 70% ethanol for 10 min, washed again for 10 min activated by US and were stored in distilled water for 24 h. The performed study protocols for the investigation of the in situ pellicle samples are well-established scientific approaches [9, 41, 42, 47–50].

### In situ investigation with selected types of bovine milk and milk proteins

A range of different commercially available types of bovine milk was used as mouthrinses in this study. Types of milk and milk proteins that in previous investigations appeared to have an effect on the ultrastructure or the composition of the in situ pellicle were included [13, 20]. Table 1 provides an overview of the selected milk types and milk protein isolates. This was to investigate possible influences of milk processing (heating, homogenization) and different content of milks’ fat (0.3%, 3.5%, 30%) on the effects of bovine milk on the bacterial biofilm formation at the tooth surface in situ. In addition to the commercially available types of bovine milk, an experimental mouthrinse was prepared from 3% native micellar casein in synthetic milk ultrafiltrate (SMUF). SMUF is a salt solution with a pH-value of 6.8. It contains KH_2_PO_4_, C_6_H_5_K_3_O_7_·H2O, C_6_H_5_Na_3_O_7_·2 H_2_O, K_2_SO_4_, KCl, CaCl_2_·2H_2_O, MgCl_2_·6H_2_O and K_2_CO_3_. The solution was prepared as described elsewhere [51].

**Table 1:** Summary of types of bovine milk and bovine milk protein isolates used in the study

Investigation samples/ Types of bovine milk & milk protein isolates	Producer
Control: no rinse/ native 8-h-biofilm sample	
UHT-treated milk 0.3% (homogenized, 0.3% fat content)	Sachsenmilch Leppersdorf GmbH, Wachau, Germany
UHT-treated milk 3.5% (homogenized, 3.5% fat content)	Sachsenmilch Leppersdorf GmbH, Wachau, Germany
Fresh milk 3.5% (homogenized, 3.5% fat content)	Sachsenmilch Leppersdorf GmbH, Wachau, Germany
Fresh milk 3.8% (non-homogenized, 3.8% fat content)	Gläserne Molkerei GmbH, Dechow, Germany
UHT-treated whipped cream (non-homogenized, 30% fat content)	K-Classic, Kaufland GmbH & Co. KG, Neckarsulm, Germany
Native micellar casein in synthetic milk ultrafiltrate (SMUF) (3% protein content)	Casein micelles were isolated from bovine milk via ultracentrifugation as described in Duerasch et al. 2018 [78]

Every participant went through all treatments and performed mouthrinses with each one of the selected types of bovine milk and bovine milk protein containing preparations. Additionally, specimens that were exposed in the mouth overnight without being rinsed were collected from every volunteer as control samples. This adds up to *n* = 7 experimental periods in total for every participant. A sequence in which the different mouthrinses were performed was randomized but similar for all volunteers. At baseline, the rinsing procedure was explained to all volunteers in detail by the laboratory staff.

All volunteers performed the different mouthrinses self-administered on different days with at least 48 h washout phase in between the experimental periods. The specific rinsing solution was prepared on the same day by the laboratory staff. No restrictions were given considering the individuals’ usual diet. One hour before insertion of the prepared oral splints, the participants had to perform oral hygiene including interdental care and at least 2 min of toothbrushing without using toothpaste or mouthrinses. Then, any further intake of foods and drinks other than water were forbidden.

The in situ exposure of the bovine enamel slabs was performed for 8 h overnight to collect samples of early bacterial biofilms. Therefore, the splints were worn in the oral cavity for 3 min to allow the adsorption of salivary components in terms of an initial in situ pellicle. Then, mouthrinses with 8 ml of one of the selected bovine milk types or milk protein isolates were performed for 3 min and the specimens’ oral exposure was continued up to 8 h until the next morning [13, 43]. Specimens that were exposed to the oral cavity for in situ biofilm formation, but without having been rinsed, served as control samples. In the morning at the lab, the slabs were removed from the splints and rinsed carefully with running tap water to remove any non-adsorbed salivary remnants [39, 41, 43]. Rinsing the biofilm with tap water is comparable to the mechanical load of rinsing the mouth with water. Further processing and analysis of the collected biofilm samples was performed in vitro.

### Fluorescent visualization of adherent bacteria and extracellular polysaccharides formation

Two of the four simultaneously intraorally exposed enamel slabs were used to visualize adherent bacteria by DAPI (4′,6-diamidino-2-phenylindole) staining [44, 52]. The fluorescent dye binds to adenine/thymidine-nucleic acids of double-stranded bacterial DNA, forming fluorescent units. Based hereon, a total count of adherent bacteria can be calculated; however, no information can be derived regarding their viability. As also described earlier, in this investigation DAPI-staining was combined with the application of Alexa Fluor 574 conjugated Concanavalin A (Invitrogen, Molecular probes, Darmstadt, Germany) to simultaneously visualize carbohydrates or extracellular matrix formation, respectively. Concanavalin A is a lectin that can bind to different types of carbohydrates [40, 42]. The working solution was prepared in a 24-well microplate from 245 µl PBS (1 mM CaCl_2_, 1 mM MnCl_2_, 1 mM MgCl_2_), 5 µl ConA-stock solution (5 mg/mL Alexa Fluor 594 conjugate in 0.1 M NaH_2_PO_4_ buffer, pH 8.3) and 0,75 µl DAPI stock solution (1 mg/ml Methanol). The enamel slabs were washed in saline solution and then incubated in the staining solution for 15 min in a dark chamber at room temperature. The solution was poured off, the slabs were rinsed carefully with saline solution and left to air-dry at room temperature. Finally, the slabs were fixed to a slide and allocated to fluorescence microscopic investigation (Axioskop II, Zeiss, Oberkochen, Germany).

All epifluorescent analyses were performed at 1000-fold magnification with a light filter for DAPI (BP 381–399, FT 416, LP 430–490) and a light filter for Concanavalin A (BP 542–576, FT 585, LP 595–664) [9, 39, 44]. Overlayering the captured images with the Zeiss-Axio-Vision software allowed the simultaneous assessment of adherent bacteria and extracellular polysaccharides. Ten representative ocular grid fields of each enamel slab were selected (1 ocular grid field = 0.0147 mm^2^). In each one, the number of cells in an area of 100 μm x 100 μm was counted and extrapolated to the number of bacteria per square centimeter. The means from all ten evaluated ocular grids were used to compare the effect of the different mouthrinses on bacterial adhesion.

For the evaluation of extracellular polysaccharides a previously described scoring system was used [48]:

#### Score 0:

No extracellular polysaccharides detectable.

#### Score 1:

single extracellular polysaccharides respectively cloudy formations.

#### Score 3:

at least 50% of the bacteria show extracellular polysaccharides.

#### Score 4:

extracellular polysaccharides structures around almost all bacteria.

### Fluorescence microscopic determination of bacteria’s viability

**Fig. 1:**
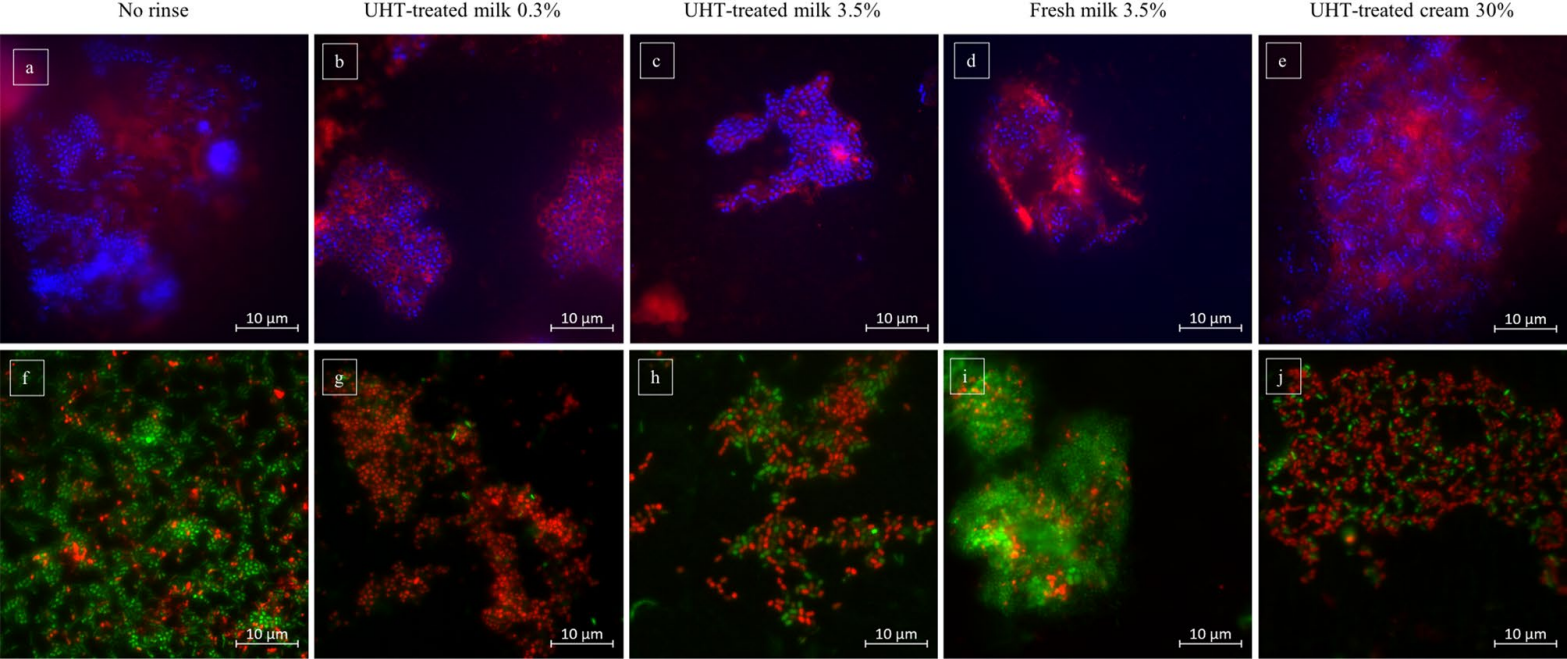
Representative images of the fluorescence microscopic investigation showing adherent bacteria (blue) and extracellular polysaccharides (red) at the enamel slabs’ surface 8 h after mouthrinses with different types of bovine milk (**b** - **e**). The control sample (**a**) showed a dense bacterial colonization embedded into an extracellular polysaccharides matrix. Mouthrinses with 0.3% UHT-treated milk (**b**) and 30% UHT-treated cream (**e**) did not seem to have an effect on the extent of biofilm formation. UHT-treated- (**c**) and fresh bovine milk (**d**) with higher fat content seemed to reduce bacteria’s adhesion and extracellular polysaccharides formation at the tooth surface. Baclight LIVE/ DEAD-staining confirmed these observations and allowed a differentiation between vital (green) and avital (red) bacteria (**f**– **j**). Compared to the control which showed a more or less equal distribution of vital and avital bacteria (**f**), all mouthrinses seemed to induce a minor shift to a higher proportion of dead bacteria (**g**– **j**)

**Fig. 2:**
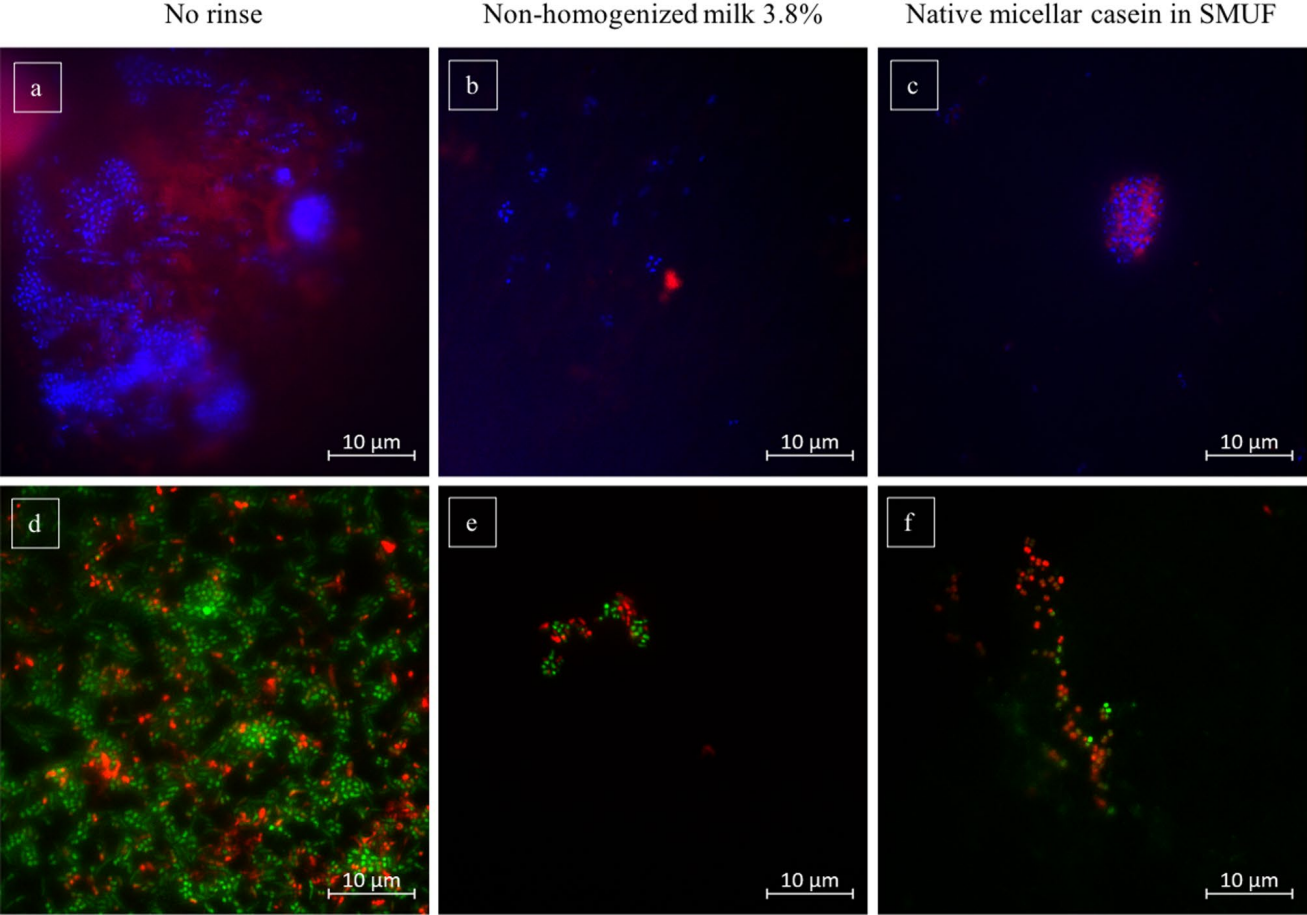
Representative images of the fluorescence microscopic investigation showing adherent bacteria (blue) and extracellular polysaccharides (red) at the enamel slabs’ surface 8 h after mouthrinses with 3.8% non-homogenized milk (**b**) or with 3% native micellar casein in SMUF (synthetic milk ultrafiltrate) (**c**). Additionally, Baclight LIVE/ DEAD staining allowed a differentiation of the adherent bacteria into vital (green) and avital (red) bacteria (**e**, **f**). After 8 h of intraoral exposure, the unrinsed controls appeared densely colonized by a thick layer of bacteria and extracellular polysaccharides (**a**). Baclight-staining showed that the high number of adherent bacteria in general (**a**) implied more or less even proportions of vital and avital bacteria (**d**). In comparison, after the application of both mouthrinses, the impression emerged that the initial bacterial colonization as well as extracellular polysaccharides formation were impaired (**b**, **c**). 8 h after the rinses with 3.8% non-homogenized milk or 3% native micellar casein in SMUF, only sparse bacterial accumulations were detected and almost no signs of extracellular matrix formation were visible (**b**, **c**, **e**, **f**)

The other two of the four simultaneously intraorally exposed enamel slabs were used to analyse the viability of adherent bacteria. The LIVE/DEAD BacLight bacterial viability assay was used to differentiate viable from dead adherent bacteria [42, 53]. One part of the staining kit, the green fluorescent SYTO 9 stain (component A) penetrates both viable and dead bacteria and binds to their DNA, while the second component, the red fluorescent propidium iodide stain (component B) (Invitrogen, Molecular probes, Darmstadt, Germany) is only absorbed by cells with a damaged cell membrane. Consequently, viable green fluorescent and dead red fluorescent bacteria can be distinguished. The staining solution was prepared from 0,5 µl of component A (Syto9 1.67 mM/propidium iodide 1.67 mM, 300 µl DMSO) and 0,5 µl of component B (Syto9 dye 1.67 mM/ propidium iodide 18.3 mM, 300 µl DMSO) in 500 µl saline solution in a 24-well microplate. After being washed, the enamel slabs were incubated with the staining solution for 10 min in a dark chamber at room temperature. The solution was poured off, the slabs were rinsed again carefully with saline solution and were left to air-dry at room temperature. The epifluorescent analysis was performed at 1000-fold magnification with a Texas Red light filter for dead bacteria (BP 542–576, FT 585, LP 595–664) and a FDA-light filter (BP 450490, FT 510, LP 515) for viable bacteria [9, 39, 52, 53]. The quantification of viable and dead bacteria was approached similarly as already described for the biofilm samples that were stained with DAPI: Ten representative areas were selected, photographed twice, viable or dead bacteria were counted on 100 μm x 100 μm and the numbers of viable and dead bacteria on 1 square centimeter were calculated. Again, the means from all 10 evaluated fields used for the comparison of the different mouthrinses. The different pictures for viable and dead bacteria were then overlapped with the Zeiss-Axio-Vision software, resulting in images like Figs. 1 and 2.

The present investigation was primarily conducted by a PhD-Student (CP) (selection of volunteers, specimens’ preparation, preparation of mouthrinses + scheduling, fluorescent staining, microscopic investigation including counting, selection of areas) and after thorough briefing and with the constant support from the laboratory staff (SB) (software, calibration etc.).

### Statistics

Statistical evaluation was carried out using the Kruskal-Wallis and the Mann–Whitney U test (*p* < 0.05). Afterwards, a Bonferroni-Holm correction took place. The used Software was SPSS 21.0 (IBM, Ehningen, Germany). The following null hypothesis was tested with DAPI, ConA and BacLight: The mouthrinses with different types of milk and milk protein have no effect on the bacterial colonization at the tooth surface in situ. Numerous previous investigations with a comparable set-up serve as valuable references [9, 40, 48, 54, 55].

## Results

### Initial bacterial colonization under the influence of milk and milk protein mouthrinses

In the present investigation DAPI-staining was used to detect all bacteria that adhere to the bovine enamel slab’s surface after they had been exposed in the oral cavity for 8 h overnight. Adherent bacteria were successfully visualized in all of the investigated samples (Figs. 1 and 2). Both intraindividual and interindividual differences regarding the amount and the distribution of detected bacteria were observed which shows consistency with several previous in situ studies of this type [9, 39, 41, 43]. The visible bacterial appearances differed from randomly spread single bacteria, to monolayered chains of coccoidal bacteria up to multilayered bacteria clusters (Figs. 1 and 2). Concanavalin A– staining was additionally applied to investigate the bacteria’s activity of extracellular matrix formation. Glucan formation by glycosyltransferases building an extracellular matrix is a relevant virulence factor of cariogenic bacteria. Concanavalin A binds at α-mannopyranosyl- and α-glucopyranosyl-residues of glucans. Representative samples of all investigated specimens showed that most of the detected adherent bacteria were surrounded by circular carbohydrate structures (Figs. 1 and 2).

**Fig. 3:**
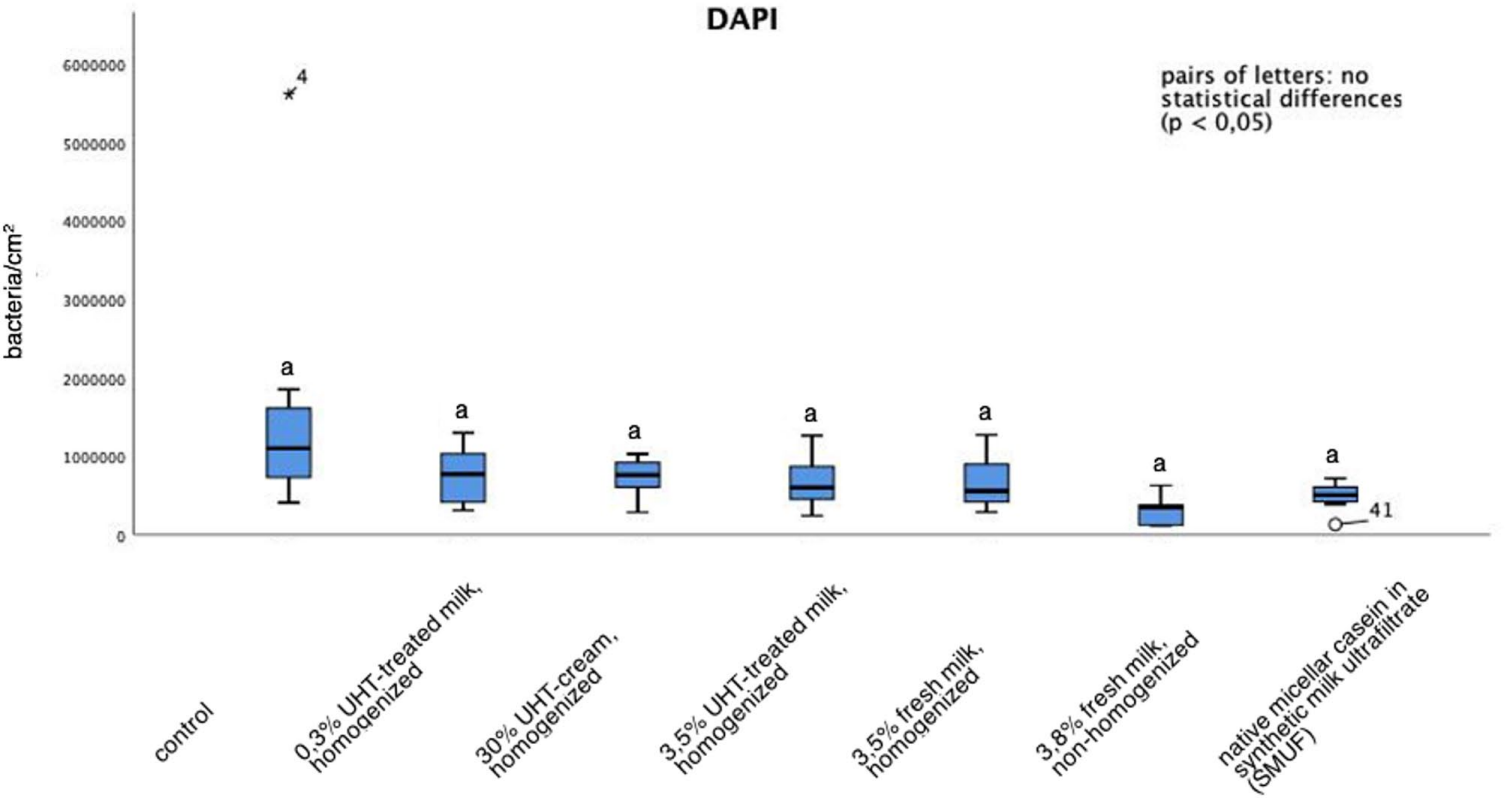
Influence of mouthrinses with different types of bovine milk and 3% native micellar casein in SMUF (synthetic milk ultrafiltrate) on the initial bacterial adhesion to bovine enamel in situ, visualized by DAPI-staining. Samples that were carried intraorally without having been rinsed served as control. All labelling was carried out in duplicate for *n* = 8 subjects. Boxplots with significant differences where marked by different letters (Mann-Whitney-U-Test and Bonferroni-Holm correction, *p* < 0.05). In comparison to the control, apparently all mouthrinses tendentially reduced the number of adhering bacteria at the tooth surface. This effect was most notable after mouthrinses either with 3.8% non-homogenized milk or after the application of a solution with 3% native micellar casein in SMUF. However, no statistically relevant variations of adhering bacteria at the tooth surface were determined between any of the performed application protocols

It clearly has to be pointed out that no statistically significant differences were proven for any of the performed mouthrinses (Fig. 3). Even if, in comparison to the control where no mouthrinse was applied (Figs. 1a and 2a), mouthrinses with certain types of milk and milk protein appeared to slightly reduce the initial bacterial colonization at the bovine enamel slabs’ surface (Figs. 1c and d and 2b and c). The observed effect appeared most striking after rinsing with non-homogenized milk (Fig. 2b) and after rinsing with the solution of native micellar casein in SMUF (Fig. 2c). However, statistical analysis did not confirm this impression. Clearly, bacterial colonization did not increase after the application of the different types of bovine milk.

For the control, a number of 1.62*10^6^ ± 1.68*10^6^ bacteria/ cm^2^ were calculated. In comparison, rinsing with non-homogenized milk reduced the number of detectable bacteria to 0.32*10^6^ ± 0.21*10^6^ bacteria/ cm^2^ and 8 h after rinsing with the experimental casein solution 0.49*10^6^± 0.18*10^6^ bacteria/ cm^2^ were quantified in the analyzed biofilm samples (Fig. 3). This was accompanied by the impression, that after mouthrinses with non-homogenized milk or native micellar casein in SMUF more single extracellular polysaccharides respectively cloudy formations were detectable which corresponds to score 1. In comparison, the control and the samples that were rinsed with any of the other mouthrinses showed distinct extracellular polysaccharides ring structures but also cloudy formations around bacteria (score 2). Yet, this observation was not statistically significant.

Even though the influence was not predominant, mouthrinses with 3.5% fresh- or UHT milk also appeared to reduce the number of adherent bacteria detectable at the tooth surface after 8 h of biofilm formation (Fig. 1c, d). In case of 3.5% fresh milk this also accounted for the visible appearance of extracellular polysaccharides structures (Fig. 1d). Neither a mouthrinse with 0.3% UHT-milk nor a mouthrinses with UHT-cream seemed to have any visible effect on bacterial adhesion or extracellular polysaccharides formation at the tooth surface (Fig. 1b, e).

### Effect of milk and milk protein on the viability of the initial bacterial biofilm

A differentiation between viable and dead bacteria in the adherent initial biofilm at the bovine enamel slabs’ surface was achieved by the simultaneous application of green fluorescent SYTO 9 stain and red fluorescent propidium iodide stain (Figs. 1f-j and 2d-f). During microscopic analysis viable green fluorescent and dead red fluorescent bacteria were detected individually spread at the tooth surface, or they were accumulated in monolayers as well as in multilayered aggregates. More dead than viable bacteria were detected in all of the investigated biofilm samples. As already shown by DAPI-staining, the application of the BacLight-viability also gave the impression that bacterial colonization of the intraorally exposed specimens was slightly reduced after mouthrinses with some of the selected types of milk and the experimental solution of micellar casein in SMUF (Figs. 1h and i and 2e and f). Again, this effect was most obvious after mouthrinses either with non-homogenized milk (Fig. 2e) or after rinsing with the solution of native micellar casein in SMUF (Fig. 2f), but not statistically significant.

**Fig. 4:**
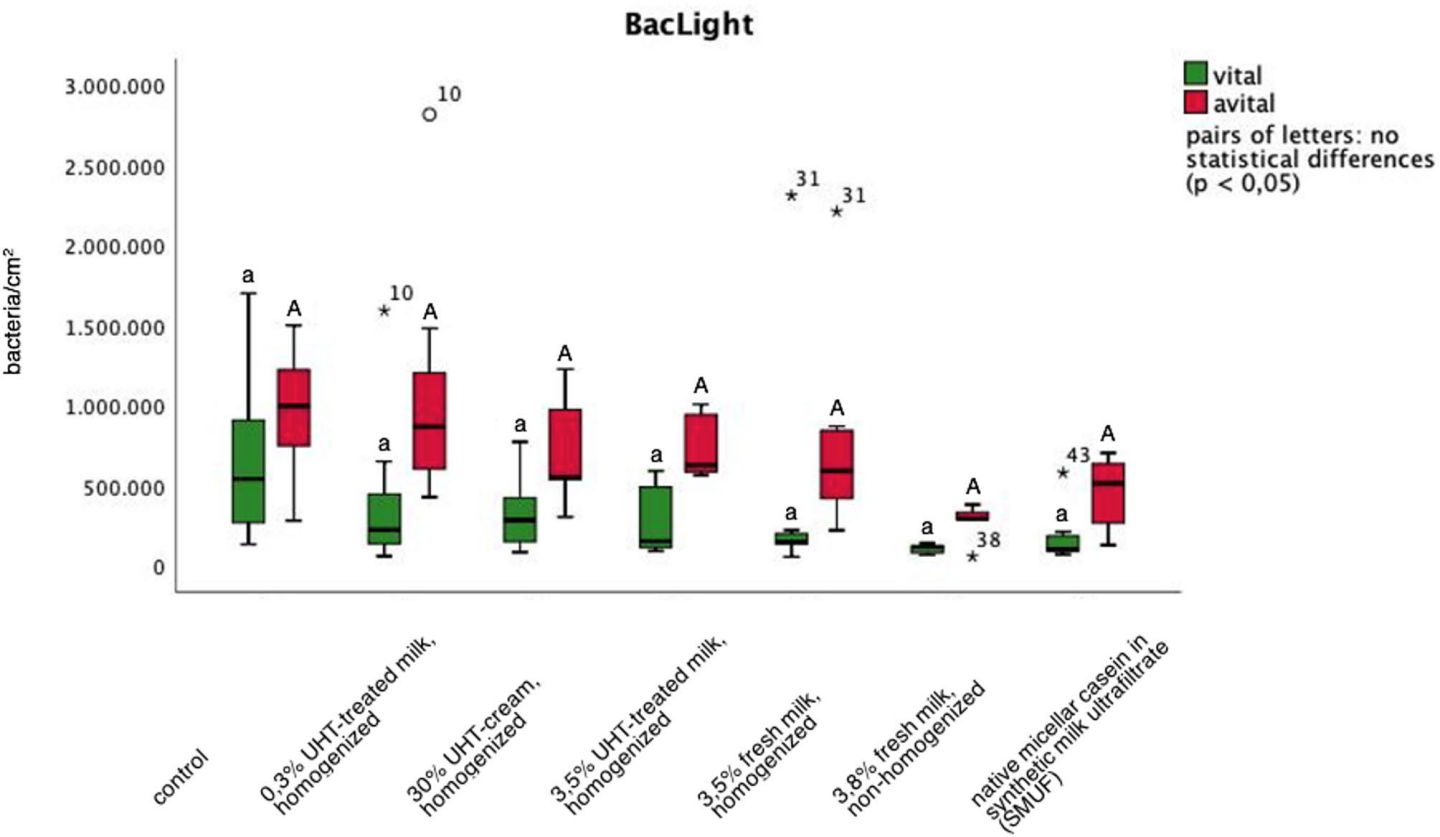
BacLight LIVE/ DEAD-staining allowed a differentiated analysis of the influence of mouthrinses with different types of bovine milk and 3% native micellar casein in SMUF (synthetic milk ultrafiltrate) on the viability of the bacteria adhering to the enamel slabs after 8 h of biofilm formation in situ (viable bacteria: green, avital bacteria: red). Samples exposed intraorally without rinsing served as control. All labelling was carried out in duplicate for *n* = 8 subjects. Boxplots with significant differences where marked by different letters (Mann-Whitney-U-Test and Bonferroni-Holm correction, *p* < 0.01). In comparison to the control, no statistically relevant influences of the mouthrinses on adhering viable or avital bacteria were detected after any of the mouthrinses. Yet, 3.8% non-homogenized milk and the solution with 3% native micellar casein in SMUF showed the tendency to reduce the number of both, viable and dead bacteria detectable at the tooth surface

Regarding the control that had not been exposed to any mouthrinses, viable and dead bacteria were distributed quite equally in the visible initial bacterial biofilm (Figs. 1f, 2d and 4): 0.66*10^6^ ± 0.52*10^6^ viable bacteria/ cm^2^ and 0.96*10^6^ ± 0.39*10^6^ dead bacteria/ cm^2^. In contrast, biofilm formation after mouthrinses with non-homogenized milk (0.10*10^6^± 0.03*10^6^ viable bacteria/ cm^2^ and 0.27*10^6^± 0.13*10^6^ dead bacteria/ cm^2^) and micellar casein in SMUF (0.18*10^6^ ± 0.17*10^6^ viable bacteria/ cm^2^ and 0.46*10^6^ ± 0.21*10^6^ dead bacteria/ cm^2^) seemed to show a slight shift towards more dead bacteria adhering to the tooth surface (Fig. 4). Yet, once again, these observations were not significant.

## Discussion

Despite the continuously growing information about the pathogenesis and prevention of oral diseases, caries and the consequences of pathogenic biofilm formation processes at the tooth surface rank among the most frequent diseases worldwide [10, 11]. Due to its omnipresence, the impact of nutrition and food components others than sugar on dental health should be investigated progressively, especially from a health-promoting perspective [1, 3, 5–7, 27, 56]. So far, little reliable information on the basis of *in vivo-* or in situ studies exists about the influence of milk and milk protein components on biofilm formation at the tooth surface [1, 12, 13, 20, 30]. This is quite astonishing, considering that in Germany alone, approximately 32.5 million tons of milk are generated per year [57]. Milk supplies several important nutrients such as iodine, calcium, proteins, vitamins and other micronutrients but its effects on human health are not fully uncritical [58]. Concerning the biofilm formation in the oral cavity, bovine milk contains the disaccharide lactose at a concentration of 3.5–5.2%. Regarding the formation of a pathogenic bacterial biofilm at the tooth surface it could be expected that the level of lactose evidently serves as a nutritional source for bacteria’s metabolism. Lactose is composed of glucose and galactose linked by a β-1.4-glycosidic bond. It is considered to be less cariogenic than other nutritional saccharides, but the application of a pure lactose-solution resulted in an increased occurrence of caries in rats [16].

It is therefore remarkable, that in the present study, no increased bacterial adhesion at the tooth surface was detected after any of the bovine milk mouthrinses. First, it is assumed that the buffering capacity of proteins contained in milk prevents a significant decrease of the pH-level at the tooth surface [4, 59]. Furthermore, there is some evidence from previous investigations that milk components and in particular micellar caseins affect the adsorption of molecules at the tooth surface and the formation of the in situ pellicle [12, 13, 20]. It was shown, that bovine milk and specifically the application of micellar caseins containing preparations temporarily influence the composition and the ultrastructural appearance of the in situ pellicle [12, 13, 20]. The adsorption of micellar caseins into the 30 min in situ pellicle was confirmed quantitatively [20]. Electrostatic interactions promote the binding of micellar caseins at the tooth surface which then might enhance further adsorption of hydrophobic proteins [29, 36, 60]. In this context, transmission electron microscopic analyses of pellicle samples that had been exposed to mouthrinses with bovine milk had detected proteins penetrating micro- and nanopores in terms of a subsurface pellicle [13, 61]. However, regarding the impact of these modifications on functional properties of the in situ pellicle, it must be acknowledge that all observed ultrastructural alterations had disappeared after 120 min of pellicle formation [13, 30].

Based on these few results from in situ studies, the present investigation now aimed to yield more information about a potential effect of ultrastructural or compositional modifications of the in situ pellicle after mouthrinses with bovine milk on the adhesion of bacteria at the tooth surface or on the formation of a viable cariogenic biofilm, respectively. Referring to the studies mentioned above, different milk components as well as different types of milk processing were considered [12, 13, 21, 30].

The performed fluorescence microscopy protocols for DAPI-, ConcanavalinA and BacLight- staining are well established techniques to analyze in situ biofilm samples [9, 42, 53, 62, 63]. Certainly, these methods do have some limitations regarding the elaborate analysis of biofilm samples: for example, a clear consideration of three-dimensional bacteria clusters is not possible. Yet, concerning the mean values of both, Dapi and BacLight staining revealed similar amounts for the total bacteria count per square centimeter, which supports the validity of the performed methods. It must be taken into account, that fluorescence microscopic approaches allow an almost immediate investigation of in situ biofilm samples with reasonable technical expenses. Of course, larger experimental groups will be necessary for an elaborate clinical study. At this point, it was intended to first get a directional idea of bovine milks’ potential to affect initial biofilm formation at the tooth surface. Clear (anti) adherent effects of a mouthrinse are detected by this approach, despite of all biological variability. Our workgroup has performed numerous studies with various substances and nutrients in this field [9, 39–41, 43, 48]. The results and the expertise collected in those studies also serve as references for the present investigation. For example, the fact that more dead than viable bacteria were detected in all of our investigated biofilm samples has repeatedly been noticed in previous comparable studies [9, 40, 41]. Certainly, antibacterial components such as lysozyme and IgA in the saliva as well as in the pellicle will be one explanation for this general observation [53]. Against this background, it is reasonable to assume, that the antibacterial components/ enzymes contained in bovine milk could enhance this effect.

Generally, the 8-h-biofilm samples were obtained overnight to reduce the impact of external influences such as a variable salivary flow or shear forces and to allow relatively reproducible conditions. An intra- and interindividual variability of the number of detectable bacteria is quite characteristic for in situ studies. Even though pellicle- and biofilm formation proceeds as a rather schematic process, interindividual differences occur regarding their thickness, specific composition or ultrastructure [64–66]. In this context, the intraoral location of the investigated biofilm, potential shear forces and the availability of components provided by the saliva are relevant influence factors.

In summary, none of the performed mouthrinses de facto influenced the initial bacterial colonization of the in situ pellicle significantly. Statistical processing of the bacterial count did not detect a significant difference between any of the analyzed biofilm samples. Clearly, no increase of bacterial adhesion was recorded after any of the different types of bovine milk mouthrinses. And that, although bovine milk provides bacterial metabolic substrate. However, even though not statistically relevant, some reduction of bacterial adherence and extracellular polysaccharide formation appeared to occur after the application of micellar caseins in SMUF and after a mouthrinse with non-homogenized milk. Considering these observations in context with the ultrastructural and compositional alteration described above, it appears reasonable that micellar caseins induce modifications of the in situ pellicle that have an influence on the expression of bacteria’s binding sites in the in situ pellicle. Also, it is assumed that casein micelles compete with glycosyltransferases for binding sites at the in situ pellicle [15, 16].

Comparing the different types of bovine milk mouthrinses, gently heated, non-homogenized milk with a lipid content of 3.8% appeared to be most likely to hamper bacterial colonization of the tooth surface.

Temperature treatment, homogenization and variable fat content of the specific types of bovine milk were considered as possible sources for different antiadhesive effects of the mouthrinses at the tooth surface. No statistically relevant differences were determined between the differently processed types of bovine milk. However, it appeared that the least processed type of milk (no homogenization, gently heated) with high fat content hampered bacterial adhesion most effectively.

The processing of milk by ultra-high temperature treatment for a few seconds at up to 140 °C in comparison to a longer exposure at max. 72 °C during pasteurization influences milk proteins’ molecular structures and properties [67]. Whey proteins denaturise at temperatures above 70 °C [68, 69]. A dissociation of micellar caseins starts at temperatures below 100 degrees depending on the type of caseins [70]. Further temperature rise promotes interactions between denaturized whey proteins and micellar κ-casein forming different protein complexes [71]. It can be concluded that heat treatment influences the proteins’ interactions and aggregation, which might also have an impact on the protein-interactions during pellicle- and initial biofilm formation at the tooth surface [72–74]. It was recognizable in the present investigation, that ultra-high temperature treated types of milk had less impact on the reduction of bacterial adhesion at the tooth surface.

However, it must be noted that a mouthrinse with pasteurised, homogenized fresh milk with a lipid content of 3.5% also appeared to have no relevant influence on the bacterial colonization of the tooth surface so that other differences between the types of bovine milk and the lipid content, respectively, should be looked at in detail.

A different antibacterial effect of pasteurized types of bovine milk could be due to the homogenisation process or the different fat content. During the process of homogenization at 15–30 MPa, milk fat droplets are split into smaller fat globules [75]. Regarding an impact of this pressure treatment on the structure or the interactions of milk proteins, it is reported in the literature that structural alterations of micellar caseins in terms of dissociation and reassociation require pressure fluctuations around 250 MPa [21, 76]. Moreover, potential molecular interactions between whey proteins and micellar caseins are only induced at pressure levels of at least 100 MPa [21]. According to the literature, it is conceivable that homogenization causes the adsorption of casein micelles into fat droplets that reassemble after being disrupted [13, 76]. Transmission electron microscopic investigations have confirmed notable ultrastructural pellicle alterations and the adsorption of casein micelles into the pellicle after mouthrinses with homogenized fresh milk of 3,5% fat content [13]: both, pellicle thickness and– heterogeneity were altered after a mouthrinse with 3.5% fresh milk. Vesicular lipid components were detected in several parts of the pellicle layer– often associated with the micellar casein aggregations. A mouthrinse with homogenized bovine milk with only 0.3% fat showed no influence on pellicle thickness or -pattern compared to the control [13]. Also, a mouthrinse with 3.8% non-homogenized bovine milk caused no remarkable alterations of the pellicle’s ultrastructure. However, looking at the results of the present investigation it must be questioned if the lipid-associated adsorption of caseins into the in situ pellicle after mouthrinses with homogenized types of UHT- or fresh milk had any hampering effect on the exposure or availability of pellicle proteins that serve as binding sites for bacteria (e.g. glycosyltransferases, amylases or prolinerich proteins and statherin). It is conceivable, that even though caseins in homogenized milk are more effectively incorporated into the pellicle, their interaction with pellicle proteins and bacterial receptor sites are influenced negatively. Also, side chains of the casein micelles could be masked by the adsorption to lipid droplets. Regarding the decrease of bacterial adhesion at the pellicle surface, non-homogenized milk with high fat content appeared to be more effective. Based on the present results after mouthrinses with non-homogenized milk and micellar caseins in SMUF it can reasonably be assumed that the natural appearance of micellar caseins is more likely to reduce bacterias’ adhesion at the pellicle surface.

## Conclusion

The present investigation contributes some more scientific data from in situ investigations to the clarification of bovine milk’s effects at the tooth surface and the results from earlier in vitro investigations do no longer apply unrestrictedly [15, 16, 19, 28]. Even though some uncertainty still remains about the specific intermolecular interactions of milk components at the tooth surface, it became clear that mouthrinses with bovine milk have no statistically significant effect on the extend of bacterial adhesion at the tooth surface. Especially the promotion of bacterial adhesion and biofilm formation at the tooth surface after milk consumption has been disproved. Specific milk components and the processing of milk influence the molecular interactions at the tooth surface. Considering the bacterial colonization of the tooth surface, non-homogenized milk with higher fat content and natural micellar caseins in SMUF appeared to have the most positive effect on the prevention of bacterial adhesion. Yet, the absence of any statistically relevant positive impact of the performed mouthrinses on the reduction of bacterial biofilm formation makes more elaborate and perhaps more costly in situ studies in this field questionable (e.g. quantitative PCR). So far, there is only little data in the scientific literature that addresses the effect of milk on in situ biofilm formation and current explanatory approaches of observed effects are still rather theoretical. Finally, this present investigation underlines the relevance of further investigations in the field of nutrients’ potential suitability to modify physiological bioadhesion processes at the tooth surface in a health-promoting manner [1, 8, 61, 77].

## Data Availability

All data associated with the present investigation is included in the manuscript. Therefore, no additional data was deposited into a publicly available repository.
